# A Music-Driven Dance Generation Method Based on a Spatial-Temporal Refinement Model to Optimize Abnormal Frames

**DOI:** 10.3390/s24020588

**Published:** 2024-01-17

**Authors:** Huaxin Wang, Yang Song, Wei Jiang, Tianhao Wang

**Affiliations:** 1 State Key Laboratory of Media Convergence and Communication, Communication University of China, Beijing 100024, China; wanghx@cuc.edu.cn (H.W.); jw@cuc.edu.cn (W.J.); wangtianhaoo@cuc.edu.cn (T.W.); 2 Key Laboratory of Acoustic Visual Technology and Intelligent Control System, Ministry of Culture and Tourism, Communication University of China, Beijing 100024, China; 3 Beijing Key Laboratory of Modern Entertainment Technology, Communication University of China, Beijing 100024, China; 4 School of Information and Communication Engineering, Communication University of China, Beijing 100024, China

**Keywords:** music-driven dance generation, spatial-temporal refinement model, abnormal frame optimization

## Abstract

Since existing music-driven dance generation methods have abnormal motion when generating dance sequences which leads to unnatural overall dance movements, a music-driven dance generation method based on a spatial-temporal refinement model is proposed to optimize the abnormal frames. Firstly, the cross-modal alignment model is used to learn the correspondence between the two modalities of audio and dance video and based on the learned correspondence, the corresponding dance segments are matched with the input music segments. Secondly, an abnormal frame optimization algorithm is proposed to carry out the optimization of the abnormal frames in the dance sequence. Finally, a temporal refinement model is used to constrain the music beats and dance rhythms in the temporal perspective to further strengthen the consistency between the music and the dance movements. The experimental results show that the proposed method can generate realistic and natural dance video sequences, with the FID index reduced by 1.2 and the diversity index improved by 1.7.

## 1. Introduction

Hearing and vision are important human sensory systems, and audio and visual signals are common modalities in life. The naturally occurring connection between the two motivates humans to learn and make inferences about audio and video and explore the correlation between these two modalities to better understand human cognition. The music-driven dance generation task implemented in this paper is a study of the multi-modal fusion of audio and video. This task focuses on mapping between music features and motion features, which can be used for dance creation in the fields of art or sports or action generation for characters in games and has great research and application potential.

Traditional music-driven dance generation methods analyze factors such as the loudness, rhythm, emotion, style, and beat of music and dance [1,2], propose similarity matrices [3], and construct motion graphs [4,5]. In addition, refs. [6,7] proposed a probabilistic framework for generating dance from music. Hidden Markov models [8] are also widely used in dance movement choreography [9,10,11,12]. Due to the excessive consideration of professional factors, the traditional methods cannot be flexibly applied to random music and dance scenes.

Recently, based on the rapid development of deep learning technologies, some deep learning-based approaches have emerged for music-driven dance generation tasks. For example, Lee et al. [13] proposed a generative method of analysis and synthesis. In the analysis stage, dance is decomposed into a series of basic dance units, and the model learns how to move through these units; in the synthesis stage, the model generates a complete dance sequence by organizing multiple basic dance movements based on the input music. Luka et al. [14] designed a Chor-RNN framework where, after training on original data, an LSTM network was utilized to achieve the prediction of dance movements. Refs. [15,16,17,18] all designed corresponding generation frameworks based on LSTM networks for generating 3D dance movements, but the generated dance sequences had the problem of abnormal movements. In order to improve the naturalness of the generated dance movements, Ren et al. [19] proposed a new pose perceptual loss in the generation stage, but the large number of motion key points still limits the quality of the synthesized dances. Yalta et al. [20] proposed to solve this kind of problem using weakly supervised learning, but the quality of the generated dances was still unsatisfactory due to a lack of human choreography experience. Sun et al. [21] proposed a cross-modal correlation framework based on GAN that aims to effectively correlate music and dance through probability. Although these approaches note the importance of specific neural network structure design for the music-driven dance generation tasks, they ignore the problem of the unnatural movements of the generated dance sequences.

**Figure 1:**
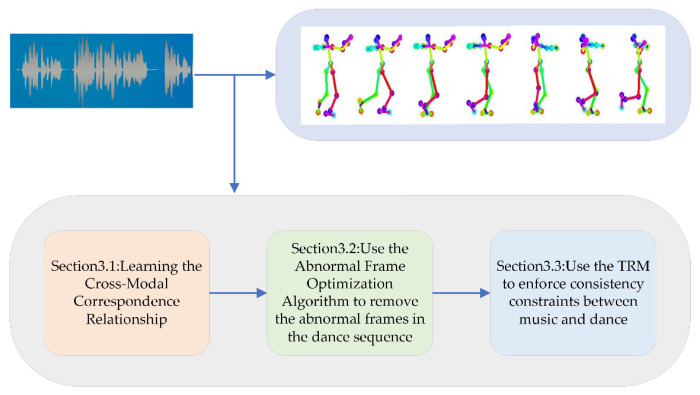
The overall flowchart of this paper is used to align the music beats and dance rhythms to further enhance the consistency between the music and the dance movements. Through spatial and temporal constraints, natural and continuous dance movements are generated.

Aiming at the problem of unnatural dance movements generated using existing deep learning methods in music-driven dance generation tasks, this paper proposes a method based on the spatial-temporal refinement model to optimize the abnormal frames, The overall flowchart is shown in Figure 1. Firstly, a cross-modal alignment model [22] is used to learn the correspondence between the audio and the dance video using pose features as an intermediary to match the best pose segments with any music based on the learned correspondence and synthesize the pose segments into a complete dance sequence. Since the pose segments are discrete, there is an abnormal motion problem between the segments. Secondly, an abnormal frame optimization algorithm is designed to solve the problem, the pixel offsets of the key points corresponding to the two adjacent frames are first calculated to locate the position of the abnormal frame and the corresponding two keyframes are determined, the keyframes are input into the spatial refinement model, and consecutive natural action frames are generated in the middle of the two keyframes and the abnormal frames are replaced. Finally, the temporal refinement model is used to enforce consistency constraints between the music and the dance.

The main contributions of this paper are summarized as follows: (1) learning the correspondence between music and dance sequences through a cross-modal alignment model to achieve the matching of optimal pose segments for any input music; (2) proposing an abnormal frame optimization algorithm, which detects the abnormal frames and carries out the optimization of the abnormal frames using the spatial refinement model; (3) aligning the music beats and the dance rhythms using the temporal refinement model to achieve the consistency constraints on the overall dance movements and music.

## 2. Related Works

In Section 2, we introduce the research works related to cross-modality generation and human movement prediction. In Section 2.1, we introduce the cross-modality generation works between text, image, audio, video, and other modalities. In Section 2.2, we introduce related works on human movement prediction, which help to understand the way dance movements are generated in music-driven dance generation tasks.

### 2.1. Cross-Modality Generation

The cross-modality generation task aims to explore the correlation between different modalities. Currently, there are extensive research works on text and images, such as image captioning [23,24] and text-to-image synthesis [25,26]. There are also works on audio and video cross-modal generation [27,28] that uses visual cues to generate background sounds for target objects in videos. Refs. [29,30] predict the motion of mouth signs from audio, focusing on speech and lip synchronization. Ref. [31] builds a hierarchical system for predicting lip movements and synthesizing speech videos. Ref. [32] trained an LSTM network on the input audio of a violin or piano performance to predict the player’s hand movements.

### 2.2. Human Movement Prediction

Movement prediction is full of randomness and spatial-temporal complexity, so dance movement generation is a challenging task. Graph convolutional networks (GCNs) can capture the spatial-temporal relationships between bodies well and extract high-level features of the human skeleton, thus in Lebailly et al. [33] the relationship between joints is learned using graph neural networks (GCNs) and the GCN architecture is combined with the initial layer of time to achieve motion prediction. The time initial layer learns long short-term information by processing the inputs of different sequence lengths. AS-GCN [34] captures richer action-specific correlations through an inference model with an encoder–decoder structure. Refs. [35,36] proposed a two-stream adaptive graph convolutional network model (2s-AGCN) based on ST-GCN and AS-GCN. This model utilizes the similarity between graph joints and adaptively learns graph structures in addition to the natural connections in the human skeleton, thus improving the accuracy of human movement recognition.

## 3. Methodology

**Figure 2:**
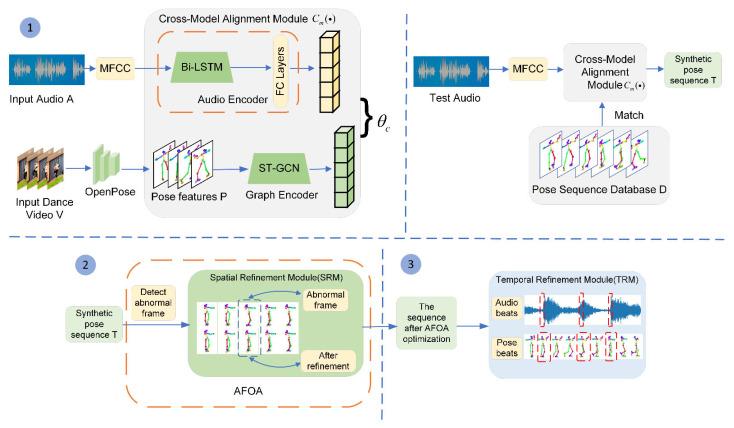
Framework diagram of the music-driven dance generation method based on the spatial-temporal refinement model to optimize abnormal frames. 1, 2, 3 in the figure denote steps 1, 2 and 3, while corresponding to the contents of Section 3.1, Section 3.2 and Section 3.3, respectively.

In this paper, we propose a music-driven dance generation method based on the optimization of anomalous frames using a spatial-temporal refinement model, as shown in Figure 2. The method consists of three main parts: (1) learning the cross-modal correspondence relationship, (2) proposing the abnormal frame optimization algorithm (AFOA), and (3) placing constraints on the consistency of the music and the dance movements. In Section 3.1, the cross-modal alignment model is utilized to learn the correspondence between audio and dance video; based on the learned correspondence, the best dance clip is matched with the input music and synthesized into a complete dance sequence. In Section 3.2, the abnormal frame optimization algorithm is proposed to solve the problem of abnormal movements in the dance sequence. In Section 3.3, the temporal refinement model is used to align the AFOA-optimized dance sequences and music to strengthen the consistency constraints between music and dance.

### 3.1. Learning the Cross-Modal Correspondence Relationship

Learning the correspondence relationship between music and dance is the key to achieving consistent audio-visual integration and is also an important step in the dance generation process. Therefore, this section uses the cross-modal alignment model [22] to learn the correspondence relationship between audio features and pose features by using the pose features as an intermediary to connect audio and dance videos. The learning process of this correspondence relationship is as follows:

Firstly, as shown in the first part of Figure 2, given an original domain paired audio A and dance video V, MFCC is used to extract the audio features of audio A and convert the frequency domain signal into low-dimensional vectors. The audio encoder consists of Bi-LSTM and FC to extract the forward and backward information of the audio to enrich the audio features. The OpenPose algorithm [37] is used to extract the pose features P of the dance video V. The pose encoder consists of an ST-GCN [38] that learns the spatial and temporal modalities of the data to extract the depth features of the pose sequence. After the feature extraction and the coding process of the audio and dance video, the audio A, dance video V, and pose feature P of the original domain can be represented as a ternary group ${\left\{x_{i}^{o}=(a_{i}^{o},v_{i}^{o},p_{i}^{o})|x\in O\right\}}_{i=1}^{N}$, where N is the total number of frames.

Secondly, the data from the original domain are input into the cross-modal alignment model $C_{m}(\cdot )$, and the correspondence between the two modalities is learned by measuring the distance between the audio features $a_{i}^{o}$ and the pose features $p_{i}^{o}$. The learning process of the correspondence can be expressed as Equation (1), where θ is the network parameter of the cross-modal alignment model $C_{m}(\cdot )$, and f(·) is the feature function.

(1) $$\theta_{C}=arg\;\underset{\theta }{\min}C_{m}\left(a_{i}^{o},p_{i}^{o}|\theta \right)f\left(i=j\right)-C_{m}\left(a_{i}^{o},p_{i}^{o}|\theta \right)f\left(i\neq j\right)$$

Finally, based on the learned correspondence $\theta_{c}$ between the audio and pose features, the best pose segments $\bar{p}$ can be matched from the original domain sequence database D (described in Section 4.1) for any target music $a^{t}$. This process can be represented as:

(2) $$\bar{p}=arg\;\underset{p\in D}{\min}C_{m}\left(a^{t},p|\theta_{c}\right)$$

### 3.2. The Abnormal Frame Optimization Algorithm

**Figure 3:**
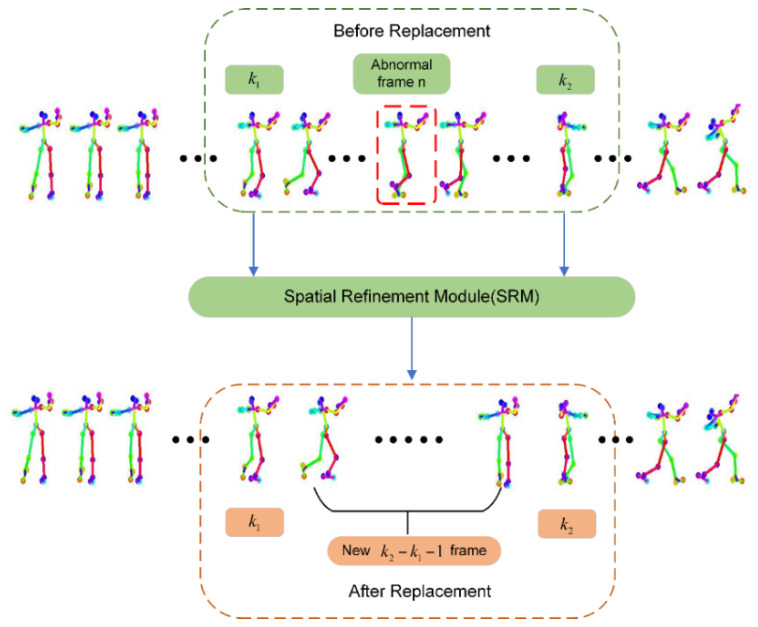
Abnormal frame optimization algorithm (AFOA) framework.

The best pose segments $\bar{p}$ obtained above are synthesized into a complete pose sequence. Since the pose segments in database D are discrete, there will be abnormal motion problems in adjacent frames at the connection between the pose segments. Therefore, in this section, the abnormal frame optimization algorithm (AFOA) is designed to solve the problem of abnormal movements and ensure the natural continuity of the dance movements. As shown in Figure 3, the AFOA includes two steps: firstly, detecting abnormal frame *n* by calculating the pixel offsets of the key points corresponding to two adjacent frames; secondly, two keyframes $k_{1}$, $k_{2}$ are determined based on the detected abnormal frame and input into the spatial refinement module (SRM), a continuous and natural pose sequence is generated between the two keyframes, and the pose segment where the corresponding abnormal frame is located is replaced. The abnormal frame optimization algorithm is introduced in two parts: detecting the abnormal frame in Section 3.2.1 and the spatial refinement model network structure in Section 3.2.2.

#### 3.2.1. Detecting the Abnormal Frame

In order to remove the abnormal frames in the dance sequence and make the dance sequence continuous and natural, the abnormal frames must be detected first. The discontinuous frames in the pose sequence are detected by calculating the pixel offsets ΔP of the key points corresponding to the two adjacent frames. The calculation of ΔP is shown in Equation (3), where $f_{1}$ and $f_{2}$ are the two adjacent frames, i are the key points labeled 0–22, and (x,y) are the pixel coordinates of the key points.

(3) $$\Delta P=\sum_{i=0}^{22}\left(|x_{f_{1}}^{i}-x_{f_{2}}^{i}|+|y_{f_{1}}^{i}-y_{f_{2}}^{i}|\right)$$

**Figure 4:**
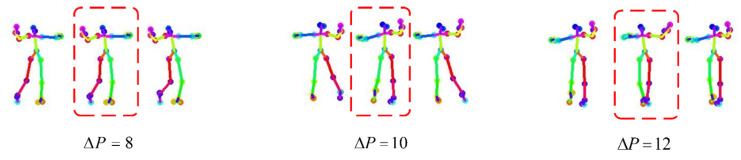
Abnormal frames in a dance sequence.

We compared the discontinuous frames detected when ΔP=8, ΔP=10, ΔP=12, as shown in Figure 4. The discontinuous frames detected when ΔP=8 were actually back-to-back frames by observation. The discontinuous frames detected when ΔP=12 have a larger mutation compared with the two frames before and after observation, so anomalous frames may be missed when setting a larger ΔP. In contrast, setting ΔP=10 can detect the discontinuous frames between the front and back frames well, so the frame with a key point movement larger than 10 pixels is set as a discontinuous frame [22].

In order to generate a continuous natural pose sequence through the spatial refinement model (SRM), two keyframes need to be set. Assuming that the detected abnormal frame is the nth frame, if $k_{1}=n-m$ and $k_{1}=n-m$ are confirmed as non-abnormal frames, then $k_{1}$ and $k_{2}$ can be defined as the keyframes. As shown in Figure 3, $k_{1}$ and $k_{2}$ input into the spatial refinement model (SRM) as keyframes generate a natural transitional dance movement sequence between $k_{1}$ and $k_{2}$, and replace the segment where the abnormal frame is located to make the movements in the whole dance sequence continuous and natural. In this experiment, we set m=3, which generates a new 5-frame movement sequence. It achieves the purpose of replacing the segment where the abnormal frame is located and controls the complexity of the network.

#### 3.2.2. Spatial Refinement Model Network Structure

**Figure 5:**
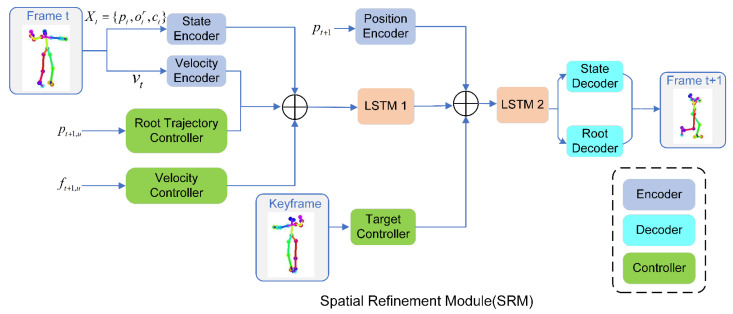
Network framework for the spatial refinement model (SRM).

In order to generate a continuous pose sequence between two keyframes to replace the pose sequence where the corresponding abnormal frame is located, this paper utilizes the spatial refinement model (SRM) [39] for movement prediction between keyframes $k_{1}$ and $k_{2}$. As shown in Figure 5, the spatial refinement model (SRM) mainly includes encoders, decoders, and controllers. The spatial refinement model uses a root trajectory controller and a speed controller based on a transformer, which can better learn inter-frame context information and achieve fine-grained motion control. The SRM uses the first LSTM to embed the pose information of the historical frame into the potential space and uses the second LSTM to predict the posture of the next frame. The following is a detailed introduction to the spatial refinement model.

The spatial refinement model (SRM) mainly consists of encoders, decoders, and controllers. Encoders: the state encoder aims to receive pose information $X_{t}=\left\{p_{t},o_{t}^{r},c_{t}\right\}$, the velocity encoder aims to receive velocity information $v_{t}$ in order to perceive the dynamics of the movement, and the position encoder aims to receive the root position information $p_{t+1}$ for the next frame. Decoders: the root decoder aims to predict the positional information of the root joint, and the state decoder aims to predict the relative position and velocity information of the other joints. Controllers: the target controller receives the pose information of the keyframes and enables the network to perceive the distance between the predicted frame and the target keyframe. The root trajectory controller and velocity controller are based on the transformer construct. The transformer structure [40,41,42] can model the dependency of sequences by using the correlation between keys and different tokens. This allows the network to capture the temporal context information of dance movement trajectories $p_{t+1,u}$ and velocity constraints $f_{t+1,u}$, and thus learn smooth context representation.

In order to enable the spatial refinement model (SRM) to generate naturally continuous dance sequences between keyframes $k_{1}$ and $k_{2}$, this paper uses four loss functions—reconstruction loss, root trajectory smoothing loss, keyframe consistency loss, and velocity consistency loss [39], to supervise the model in an end-to-end form. These four loss functions will be introduced separately below.

(1) Reconstruction Loss: The reconstruction loss is constructed by the mean square error (MSE) loss to measure the similarity between the predicted movement and the real movement and motivates the network to generate a motion sequence that satisfies the constraints. The reconstruction loss is shown in Equation (4), where n is the length of the sequence, $X_{t}=\left\{p_{t},o_{t}^{r},c_{t}\right\}$ represents the true value information at time t, including the root position information $p_{t}$, the rotation angle $o_{t}^{r}$ of the root, and the velocity factor $v_{t}$.

(4) $$L_{rec}=\frac{1}{n}\sum_{t=k_{1}+1}^{k_{2}}{\left\|{\hat{X}}_{t}-X_{t}\right\|}^{2}$$

(2) Root Trajectory Smoothing Loss: It enhances temporal consistency by minimizing the differences in the spatial position and rotation angle of the root joint between frame t and frame t−1. The root trajectory smoothing loss is shown in Equation (5):

(5) $$L_{root}=\frac{1}{n}(\sum_{t=k_{1}+1}^{k_{2}}{\left\|p_{t}-p_{t-1}\right\|}^{2}+\sum_{t=k_{1}+1}^{k_{2}}{\left\|o_{t}^{r}-o_{t-1}^{r}\right\|}^{2})$$

(3) Keyframe consistency loss: The purpose of the spatial refinement model is to generate natural transitional dance movements between two keyframes, which requires ensuring the continuity of the predicted movements near the keyframes. The keyframe consistency loss is shown in Equation (6), where s is the number of frames affected by the keyframe, refer to [39] to set s=3.

(6) $$L_{key}=\frac{1}{2s}(\sum_{t=k_{1}+1}^{k_{1}+s}{\left\|{\hat{p}}_{t}-p_{k_{1}}\right\|}^{2}+\sum_{t=k_{2}-s+1}^{k_{2}}{\left\|{\hat{p}}_{t}-p_{k_{2}}\right\|}^{2})$$

(4) Velocity consistency loss: It ensures that the velocity of the synthesized dance sequence is consistent with the given control conditions, as shown in Equation (7), where $f_{t}$ is the velocity constraint given according to the overall dance sequence, and ${\tilde{f}}_{t}$ is the velocity of the calculated predicted dance movements.

(7) $$L_{vfac}=\frac{1}{n}\sum_{t=k_{1}+1}^{k_{2}}{\left\|{\tilde{f}}_{t}-f_{t}\right\|}^{2}$$

The complete loss function is shown in Equation (8), where $\omega_{rec}$, $\omega_{root}$, $\omega_{key}$ and $\omega_{vfac}$ are the corresponding loss weights, set to 0.3, 0.15, 0.2, and 0.15, respectively.

(8) $$L=\omega_{rec}L_{rec}+\omega_{root}L_{root}+\omega_{key}L_{key}+\omega_{vfac}L_{vfac}$$

### 3.3. Music and Dance Consistency Constraints

After processing using the spatial refinement model, the abnormal movements in the dance sequences are well smoothed from the spatial perspective. But overall, there will be a phenomenon where the music beat is inconsistent with the dance rhythm. Therefore, the temporal refinement model [22] is used to further constrain the music and dance from a temporal perspective. The constraint is to align the music beat with the dance rhythm.

Firstly, the beats of the target audio $a^{t}$ are extracted using the Librosa library [43], and the dance rhythms are defined as postural movements with large variations, as shown in Equation (9):

(9) $$d_{\mu }=arg\;\underset{j}{\max}\left(|p_{j}^{t}-p_{j-1}^{t}|\right),\;j\in \left\lceil i,\right.\left.i+\omega_{c}\right\rfloor$$

Secondly, a sliding window of $\omega_{c}$ frame size is set to search the local maximum to align. After the detected music beat $a_{\mu }$, the $\omega_{c}$ frames can be reorganized as $\left[i,\mu \right]$ and $\left(\mu ,\right.\left.i+\omega_{c}\right]$. For the preceding and the following frames of the current beat point, the cubic fitting interpolation method is adopted to obtain the aligned pose ${\hat{p}}^{t}$. The alignment function is denoted as:

(10) $$a^{*}=arg\;\underset{a}{\min}\sum_{x=i}^{\mu }{\left(\sum_{m=0}^{3}a_{m}x^{m}-p_{x}^{t}\right)}^{2}$$

(11) $${\hat{p}}^{t}=a_{0}^{*}+\sum_{m=1}^{3}a_{m}^{*}x^{m}$$

**Figure 6:**
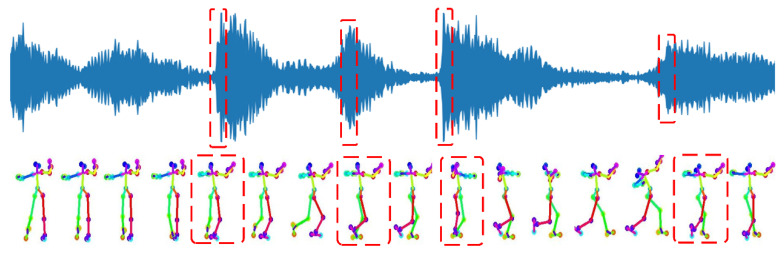
Temporal refinement model aligning music beats and dance rhythms. The first row of red dashed boxes indicates the detected music beats and the second row of red dashed boxes indicates the dance rhythms.

After the optimization of the temporal refinement model, as shown in Figure 6, the music beats and dance rhythms can be aligned well, and the music and dance movements can be better integrated as one.

## 4. Experiments

In this section, we first describe the dataset used in the experiment and the implementation details. Then, the proposed method is compared with other methods, and ablation studies using different optimization strategies are conducted to prove the effectiveness of our proposed method. Finally, the dance sequences generated by different methods are visualized for subjective evaluation and comparison.

### 4.1. Dataset and Implementation Details

The experiments in this paper are performed on the dataset of Guo et al. [22], which collects dance videos from real scenarios and can obtain the dancer’s movements and audio at the same time. The dataset contains 122 female dance videos and 32 male dance videos and each video length ranges from 3 to 5 min, totaling 9 h. The resolution of the videos is 1920 × 1080 and is processed at a standard frame rate of 24 fps, and the size of the dance image extracted from the frame is adjusted to a uniform size. The OpenPose algorithm [37] is used to extract human key points from the dance images. Each pose consists of 23 joint points. The frames whose key points are not fully detected and abnormal frames with sudden motion changes are deleted. After processing the abnormal data, the pose sequences are intercepted into different pose segments and finally constructed as pose segment database D.

To evaluate the quality of the generated pose sequences, 80% of the dataset was randomly selected for training and the remaining 20% for testing. The model in this paper was implemented using PyTorch on an RTX-3090 GPU and trained for 500 epochs. The spatial refinement model was trained using the Adam optimizer with a learning rate of 0.001 and batch size of 128.

### 4.2. Evaluation Metrics

(1) FID: Fréchet inception distance (FID) is a commonly used evaluation index in image processing, which can effectively calculate the feature distance between the real samples and the generated samples. The smaller the FID value, the closer the generated data is to the real data, and the better the effect of the model. The calculation of FID is shown in Equation (12), where Trace is the trace of the matrix, m and C are the mean and covariance of the probability distribution of the generated data, respectively, and $m_{T}$ and $C_{T}$ are the mean and covariance of the probability distribution of the real data, respectively.

(12) $$d^{2}\left(\left(m,C\right),\left(m_{T},C_{T}\right)\right)={\left\|m-m_{T}\right\|}_{2}^{2}+Trace(C+C_{T}-2{\left(CC_{T}\right)}^{1/2})$$

(2) Beat Hit Rate: $R_{hit}$ is the ratio of the number of motion beats aligned with music to the total number of motion beats, $R_{hit}$ calculated as shown in Equation (13). The total number $B_{K}$ of motion beats and the number $B_{A}$ of motion beats aligned with the music beats is counted. If the input music is synchronized with the generated dance movements, the indicator $R_{hit}$ will be higher.

(13) $$R_{hit}=\frac{B_{A}}{B_{K}}$$

(3) Diversity and multimodality: Diversity refers to the differences between the generated dance movements, the greater the differences, the greater the diversity. The diversity is calculated by randomly sampling the 40 dances generated and calculating the average FID value between them. The larger the value, the greater the differentiation between all the movements, and the greater the diversity of the dances. Multimodality refers to the ability to generate different dances given the same musical conditions, demonstrated through visualization.

### 4.3. Comparison of the Results to Other Methods

This paper compares the methods of Lee et al. [13] and Guo et al. [22] using four metrics: FID, beat hit rate, diversity, and multimodality.

The results of comparing this paper’s method with the methods of Lee et al. [13] and Guo et al. [22] are shown in Table 1. The dance sequence generated using this paper’s method has the smallest FID value, and the generated dance sequence is closer to the real values. The increase in beat hit rate indicates that the consistency between the generated dance rhythms and the music beats is improved, indicating that the temporal refinement model imposes an effective consistency constraint on the music and dance. The diversity metric is improved by 1.7, indicating that compared with the traditional TSD algorithm used by Guo et al. [22], the spatial refinement model based on LSTM and the transformer proposed in this paper can generate rich dance movements. Correspondingly, the multimodal metric is also improved, further proving that the method in this paper can generate rich dance movements.

### 4.4. Ablation Study

In this section, the ablation experiments of the three processing methods for abnormal frames are compared: no abnormal frame optimization processing, processing based on the traditional TSD algorithm, and processing based on the AFOA algorithm proposed in this paper. As shown in Table 2, it can be seen through the results that the experimental results of no abnormal frame optimization processing are the worst; after Guo et al. [22] optimized the abnormal frames using the traditional TSD algorithm, the FID value and diversity are improved. The experimental results of the AFOA algorithm proposed in this paper are the best, indicating that the optimization strategy of generating continuous natural dance movements through the spatial refinement model between keyframes $k_{1}$ and $k_{2}$ is effective. It ensures the natural continuity of dance movements and improves the diversity of dance movements.

### 4.5. Visualization of the Generated Dance Sequence

**Figure 7:**
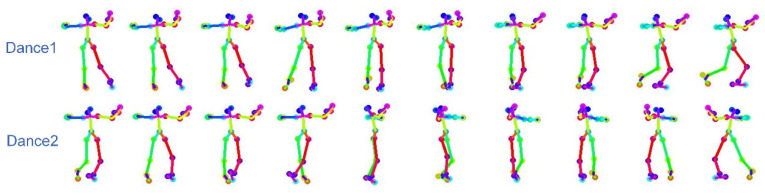
Multimodality: given the same musical conditions two different dance sequences can be generated.

**Figure 8:**
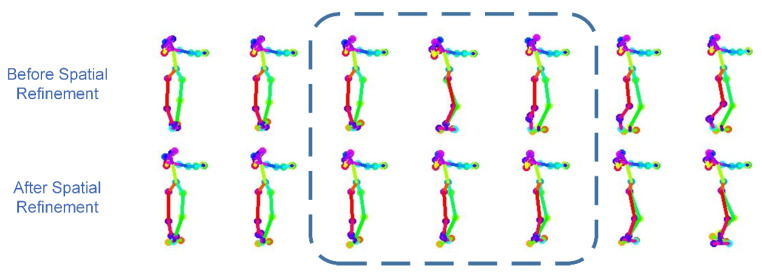
The spatial refinement model optimizes abnormal dance movements.

The generated dance sequences are visualized for subjective evaluation and comparison. As shown in Figure 7, two different dance sequences can be generated given the same music, reflecting the multimodality of the method in this paper. As shown in Figure 8, the comparison before and after the spatial refinement model optimizes the abnormal frames. Through the visualization results, it can be seen that after the spatial refinement model optimizes the abnormal frames, the dance movements can be made natural and continuous.

**Figure 9:**
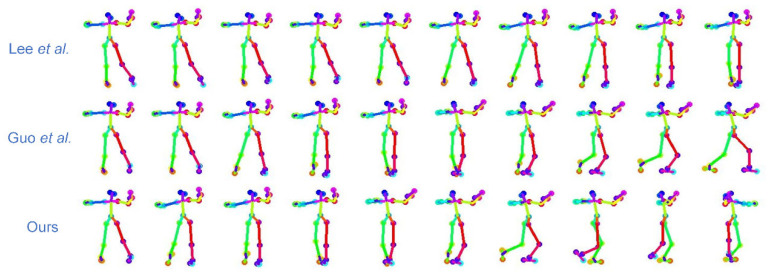
Visual comparison between the method of this paper and the methods of Lee et al. [13] and Guo et al. [22].

The dance sequences generated using the method of this paper can be visually compared with the methods of Lee et al. [13] and Guo et al. [22]. As shown in Figure 9, the dance movements generated by Lee et al.’s method have fewer changes and the dance diversity is poor. In contrast, Guo et al.’s method has significant improvements, but the generated movements also have abnormal jitter. The abnormal frame optimization algorithm (AFOA) proposed in this paper generates natural and continuous pose segments between two keyframes through the spatial refinement model (SRM). It achieves the purpose of optimizing abnormal frames, making dance movements continuous and natural, and improving the diversity of dance movements.

## 5. Conclusions and Discussion

Aimed at the problem of dance movement mutation in existing music-driven dance generation methods when generating dance sequences, this paper proposes a method to optimize abnormal frames based on a spatial-temporal refinement model. The method consists of three parts: (1) The cross-modal alignment model is used to learn the correspondence between audio and dance video modalities, match the best dance segments for any input music based on the learned correspondence, and synthesize it into a complete dance sequence. (2) Since the dance segments are discrete, there is a mutation problem in the dance movements between segments. In order to solve this problem, the abnormal frame optimization algorithm is proposed. First, by calculating the pixel offset of the corresponding key points of two adjacent frames, the positions of the abnormal frame and the corresponding two keyframes are determined. Then, the spatial refinement model generates a natural transition pose sequence between the two keyframes and replaces the pose sequence where the corresponding abnormal frame is located to optimize the abnormal frame. (3) The temporal refinement model is used to align the music beats and dance rhythms to enhance the consistency of the music and dance movements from the temporal perspective. Experiments have shown that the method proposed in this paper can generate dance sequences with natural and continuous movements.

Based on the music-driven dance generation method proposed in this paper, a virtual reality-based real-life dance performance system can be designed. The stage combined with virtual reality technology can bring novel and intuitive artistic audio-visual experience to the audience. The system can assist professionals in choreographing dance movements and can also provide dance movement instruction for amateurs who love to dance. 

Packaging the demo system as a software tool can make the system easier to deploy in more application scenarios. In future work, we plan to collect and integrate music genres, dance styles and other factors to make the generated dance more delicate and relevant to the theme.

## Figures and Tables

**Table 1:** Comparison of objective indicators of dance generation between the method proposed in this paper and other methods. **↓** indicates that larger values are better, and **↑** indicates that smaller values are better.

Method	FID ↓	Beat Hit Rate ↑	Diversity ↑	Multimodality ↑
Real dances	5.9	51.6%	53.5	-
Lee et al. [13]	12.8	65.1%	53.2	47.8
Guo et al. [22]	8.6	70.3%	58.6	57.8
Ours	7.4	72.6%	60.3	58.3

**Table 2:** Ablation studies with different optimization strategies. **↓** indicates that larger values are better, and **↑** indicates that smaller values are better.

Method	FID ↓	Diversity ↑
Real dances	5.9	53.5
No abnormal frame optimization process	11.2	56.2
Guo-TSD	8.6	58.6
Ours-AFOA algorithm	7.4	60.3

## Data Availability

Datasets during the current study are available from https://drive.google.com/file/d/1vPyOqaIT-nmB5Yb8HQ0FZk8Usg2RD8Vp/view (accessed on 21 November 2023).
